# Platelets induce VISTA expression and modulate the ovarian tumor microenvironment

**DOI:** 10.1080/09537104.2026.2644366

**Published:** 2026-03-17

**Authors:** Hani Lee, Brianne Sager, Anil Sood, Vahid Afshar-Kharghan, Min Soon Cho

**Affiliations:** 1 The Section of Benign Hematology, The University of Texas MD Anderson Cancer Center, Houston, TX, USA;; 2 The Department of Gynecology Oncology & Reproductive Medicine, The University of Texas MD Anderson Cancer Center, Houston, TX, USA

**Keywords:** Immune checkpoint, neutrophils, ovarian cancer, platelet

## Abstract

Immune checkpoint regulators, such as the V-domain Ig suppressor of T cell activation (VISTA), play a critical role in shaping the tumor microenvironment (TME) and facilitating immune evasion. In ovarian cancer, VISTA exhibits more abundant and consistent expression than other immune checkpoints, including Programmed Death-Ligand 1 (PD-L1). This study examined the role of platelets in the regulation of VISTA in ovarian cancer using both in vitro and in vivo models. Our findings demonstrate that platelets upregulate VISTA expression in both myeloid and tumor cells, thereby promoting an immunosuppressive TME. Elevated VISTA levels were associated with higher platelet counts and poorer clinical outcomes. These results highlight that platelet-mediated VISTA upregulation is a potential therapeutic target for improving antitumor immune responses in ovarian cancer.

## Introduction

Ovarian cancer remains a leading cause of gynecologic cancer-related deaths^1^, marked by a profoundly immunosuppressive tumor microenvironment (TME). Despite advances in treatment, the prognosis of advanced ovarian cancer remains poor, primarily due to chemotherapy-refractory relapses and resistance to immunotherapy.^2,3^ The interplay between cancer cells, immune cells, and stromal cells within the TME shapes the immune response and drives disease progression.^4,5^ Immune checkpoint proteins, such as PD-L1 and V-domain Ig suppressor of T cell activation (VISTA), are central to this regulatory network, suppressing antitumor immunity.^6^ We have previously shown that platelets contribute to immune modulation within the TME, upregulate PD-L1 expression, and influence the efficacy of anti-PD-L1 immunotherapy in murine models of ovarian cancer.^7^

VISTA has recently emerged as a critical regulator of immune suppression in the TME, particularly through its activity on myeloid cells, which inhibits T-cell activation and facilitates immune evasion.^8–13^ In high-grade serous ovarian cancer (HGSOC), VISTA exhibits broad and consistent expression,^14,15^ especially in tumor-infiltrating myeloid cells, whereas PD-L1 expression is heterogeneous and generally low to moderate. While PD-L1 is predominantly expressed on immune cells and infrequently on tumor epithelial cells, VISTA is more abundantly and uniformly expressed across the tumor microenvironment^14,16^ and even in PD-L1-negative tumors.^16^ These differences suggest that VISTA may represent a more relevant and consistently expressed immune checkpoint target than PD-L1 in ovarian cancer.

Beyond their established role in hemostasis, platelets are increasingly being recognized as active participants in cancer progression.^17^ They protect circulating tumor cells from immune surveillance, promote their adhesion to the vascular endothelium, and secrete growth factors that enhance tumor survival, proliferation, and metastasis.^18^ By shielding tumor cells and modulating immune cell function, platelets foster an environment that supports tumor growth and impairs effective immune responses.^19–21^ In ovarian cancer, platelets not only promote metastatic dissemination,^17,22,23^ but also, as demonstrated by our group, enhance cancer cell proliferation and the growth of primary tumors.^24–26^ In addition, several studies have shown that platelets upregulate PD-L1 expression, further reinforcing the immunosuppressive nature of the tumor microenvironment (TME).^27,28^ However, the effect of platelets on VISTA expression has not yet been explored. In this study, we investigated the role of platelets in the regulation of VISTA expression in ovarian cancer and myeloid cells. Our goal was to identify novel therapeutic targets that can disrupt platelet-mediated immune suppression, thereby enhancing antitumor immunity and improving outcomes in patients with ovarian cancer.

## Materials and methods

All mouse studies were carried out using protocols approved by the Institutional Review Board (IRB) and the Institutional Animal Care and Use Committee of the University of Texas MD Anderson Cancer Center (UT-MDACC). Deidentified tumor blocks were used under an IRB-approved protocol, and informed consent was not obtained.

### Antibodies

The list of antibodies used in our study, along with relevant information including vendors, catalog numbers, and RRIDs, is provided in Supplementary Table 1.

### Animal experiments

Female C57BL/6 mice were purchased from Jackson Laboratory. For tumor induction, 8-week-old mice were intraperitoneally injected with 2 × 10 ID8 cells (murine ovarian cancer cells derived from C57BL/6 mouse ovarian surface epithelium) suspended in 200 μL of Hank’s balanced salt solution (HBSS). One week before injection, mice were administered either an antiplatelet antibody (APA) via tail vein injection or recombinant thrombopoietin (TPO) via subcutaneous injection to assess the impact of platelets on tumor growth. Mice were monitored for 4–5 weeks for general well-being and tumor growth until they became moribund. For experiments involving platelet depletion, monoclonal anti-GPIb-α antibody (R300) was administered via tail vein injection at a dose of 0.5 μg/g twice weekly, and TPO (recombinant murine thrombopoietin) was delivered subcutaneously at 10 μg/kg once weekly; both treatments were continued until the tumor-bearing mice became moribund. Upon reaching a moribund state, mice were euthanized in accordance with institutional animal care guidelines. Tumors were excised, weighed, and processed for sample preparation according to the experimental purpose. In a group of mice, an anti-mouse PD-L1 antibody or an isotype-matched control antibody was injected intraperitoneally on days 2 and 4 after the injection of murine ovarian cancer cells.

### Isolating platelets and PBMC from donors

Platelets were isolated from the peripheral blood collected from healthy donors (with informed consent) and ovarian cancer patients under IRB-approved protocols. Whole blood was anticoagulated with acid-citrate-dextrose (ACD; 1:9 v/v) and centrifuged at 200 × g for 15 min without brake to obtain platelet-rich plasma (PRP). The PRP was subsequently centrifuged at 150 × g for 10 min to pellet the remaining red blood cells, followed by centrifugation at 600 × g for 5 min to pellet the platelets. The platelet pellet was gently resuspended in Tyrode’s buffer, and the washed platelets were counted using a hemocytometer. All experiments were performed using washed platelets. The resting state of washed platelets was confirmed by comparing the expression of the active conformation of GPIIb/IIIa before and after thrombin stimulation (Supplementary Figure 1).

Peripheral blood mononuclear cells (PBMCs) were isolated by density gradient centrifugation with Ficoll. Whole blood collected in EDTA tubes was diluted 1:1 with DPBS and carefully layered over Ficoll in 50 ml conical tubes. Samples were centrifuged at 450 × g for 30 minutes at room temperature with the brake off. The mononuclear cells were collected and washed twice with DPBS containing 2 mM EDTA by centrifugation at 300 × g for 10 minutes to remove residual platelets. PBMC were then counted using acridine orange/propidium iodine (AO/PI) staining and resuspended in culture medium for subsequent experiments.

### Ovarian cancer cell culture

Human ovarian cancer cells Hey A8 (CVCL_8878), SKOV3 (CVCL_0134), OVCAR 5 (CVCL_1628), and OVCAR8 (CVCL_1629) were cultured in RPMI-1640 medium supplemented with 10% fetal bovine serum (FBS) and 1% penicillin-streptomycin at 37°C in 5% CO_2_. Murine ovarian cancer cells ID8(CVCL_IU14) were maintained in DMEM medium supplemented with 10% fetal bovine serum and 1% penicillin-streptomycin. Cancer cells (1 × 10^5^/well) were seeded in 6-well plates overnight, washed twice with PBS, and co-incubated with washed platelets (1:20 ratio) in serum-free RPMI-1640 for 24 h at 37°C with 5% CO_2_.

### Cancer cell-platelet co-culture

Cancer cells (1 × 10^5^/well) were seeded in 6-well plates and allowed to adhere overnight, washed twice with PBS, and co-incubated with washed platelets at a 1:20 (cancer cells:platelets) ratio (2 × 10^6^ platelets/well) in complete culture medium for 24 h at 37°C with 5% CO_2_.

### PBMC-Platelet co-culture

For immune cell co-culture experiments, PBMC (2 × 10^6^ /well) were seeded in 24-well plates and incubated with platelets at a 1:20 ratio (4 × 10^7^ platelets/well) in complete culture medium. After 24 hours, Cells were stained with a viability dye and fluorochrome-conjugated antibodies to identify immune cell subsets and assess VISTA expression. Samples were analyzed on a BD Fortessa X-20 or a Cytek Aurora spectral cytometer.

### Flow cytometry analysis

PBMCs were isolated after red blood cell lysis and resuspended in staining buffer (PBS with 2% FBS). Cells were stained with fluorophore-conjugated antibodies for 20 min at 4°C, washed twice, fixed in 1% paraformaldehyde, and analyzed on a Cytek Aurora flow cytometer. Data analysis was performed using the FlowJo software. Based on forward and side-scatter parameters, cells were gated to exclude debris and doublets. Further gating was performed to identify specific cell populations, including B cells (CD19+), CD4 T cells (CD4+), CD8 T cells (CD8+), and monocytes (CD14+ in humans; CD11b+ Ly6C+ Ly6G- in mice). VISTA expression was analyzed within each gated population, and values were normalized to the mode for graphical representation. The gating strategy for flow cytometry is shown in Supplementary Figure 2. tSNE plots were generated in FlowJo software from the gated populations stained for CD45, CD3, CD19, CD11b, CD14, CD16, and VISTA.

### Immunohistochemistry

Immunohistochemistry (IHC) staining for VISTA was conducted on 4-μm-thick sections of formalin-fixed, paraffin-embedded tumor nodules using previously established methods.^25^ Briefly, tumor sections were first deparaffinized. Antigen retrieval for VISTA was performed in a humid steam chamber using citric acid buffer (pH 6). Tissue sections were treated with 3% H_2_O_2_ in PBS to block nonspecific binding and blocked with 5% donkey serum. They were then incubated with primary anti-VISTA antibody for 1 hour at room temperature, followed by washing. The slides were subsequently incubated with an anti-rabbit horseradish peroxidase (HRP)-conjugated secondary antibody for 1 hour, then exposed to the HRP substrate. The intensity of IHC staining and the percentage of tumor surface area stained in tumor tissues were evaluated using densitometry in ImageJ software.

### Western blot analysis

Cells were lysed in RIPA buffer supplemented with protease and phosphatase inhibitors, homogenized using an insulin syringe, and incubated on ice for 30 min. Lysates were centrifuged at 16,000 × g for 30 min at 4°C, and the supernatants were collected. The protein concentration was measured using a BCA assay. Protein samples were mixed with 4 X sample buffer and denatured at 95°C for 10 minutes. Proteins were separated by SDS-PAGE and transferred to PVDF membranes, which were then blocked in a 5% blocking solution for 30 minutes at room temperature. The membranes were incubated overnight at 4°C with primary antibodies, followed by 1 hour at room temperature with HRP-conjugated secondary antibodies. Protein bands were visualized using chemiluminescent substrates and detected using a Bio-Rad ChemiDoc system. The intensity of protein bands on the immunoblots was quantified using densitometry in ImageJ.

### Single-cell RNA sequencing data analysis

Publicly available single-cell spatial transcriptomics data for high-grade serous ovarian cancer (HGSC) were obtained from the Broad Institute Single Cell Portal (https://singlecell.broadinstitute.org/single_cell), with portal ID: SCP2640. The dataset comprises processed spatial transcriptomic profiles for 2.5 million cells in situ from 130 tumors across 94 patients. We downloaded the transcript per million (TPM) expression matrix and the corresponding metadata with cell-type annotations. As the original authors had preprocessed the dataset, no additional cell-level filtering was performed in this study.

All Analyses and visualizations were performed in Seurat and ggplot2. *VSIR* expression was extracted and visualized in a UMAP embedding using FeaturePlot and DimPlot, with customized ggplot2 overlays.

### Statistical analysis

Laboratory parameters were described using summary statistics, including means and medians (interquartile ranges). VISTA levels were analyzed using the Mann-Whitney U test, as the data were non-parametric. Welch’s t-test was used to compare means. Statistical significance was set at *p* < .05. Correlations between parameters were assessed using Spearman’s correlation test. Due to the limited number of patients in the exploratory study, a multivariate analysis could not be conducted. Statistical analyses were performed using GraphPad Prism 10.

## Results

### Platelets increase VISTA expression in ovarian cancer cells *in vitro*

**Figure 1.**
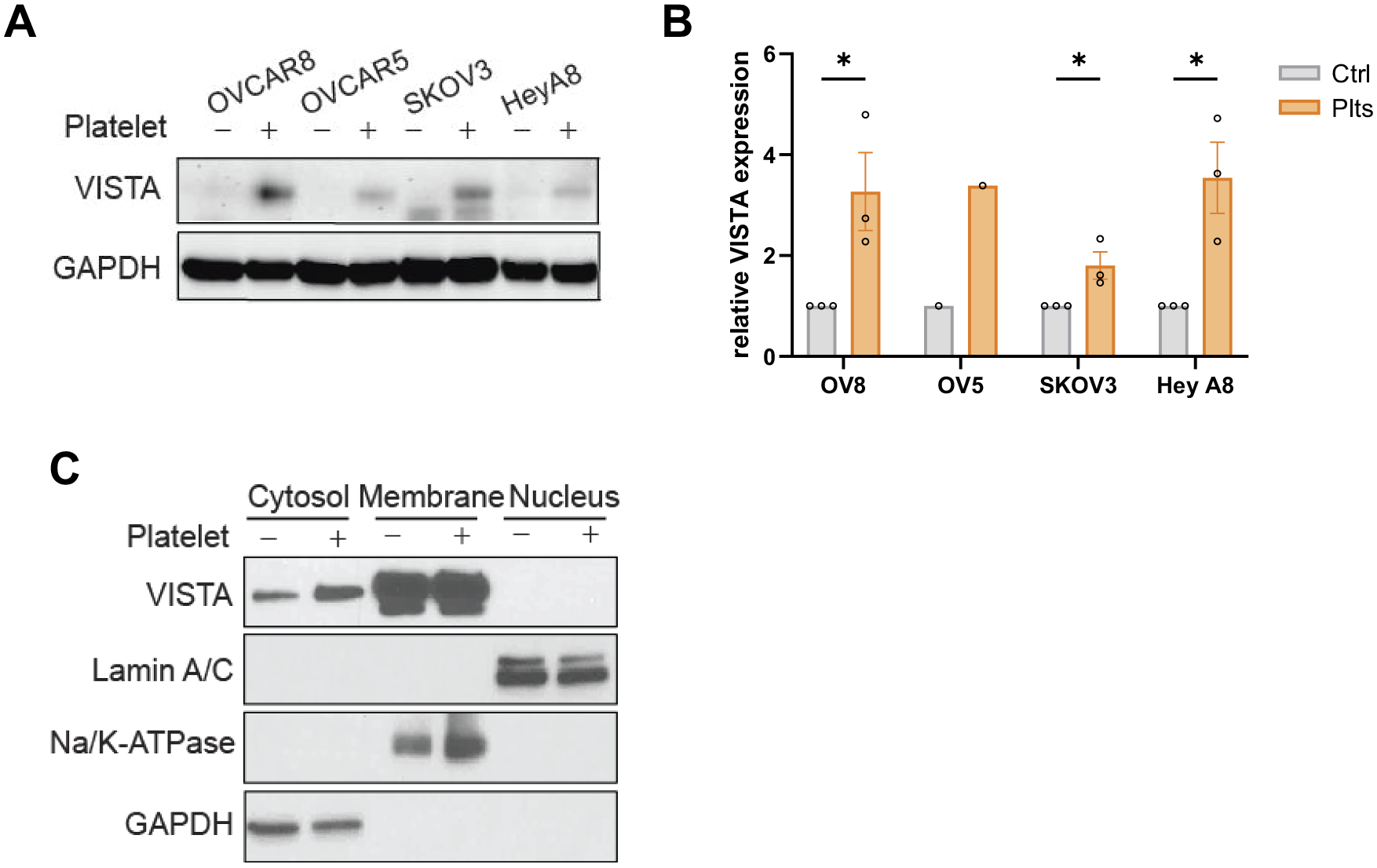
Platelets increase VISTA expression in ovarian cancer cells *in vitro*. (A) Western blot analysis showing platelet-induced VISTA expression across various ovarian cancer cell lines (OVCAR8, OVCAR5, SKOV3, and HeyA8). GAPDH served as a loading control. (B) The relative increase in VISTA expression in ovarian cancer cells following incubation with platelets was quantified by densitometric analysis of Western blot bands (*n* = 2–3 blots per cancer cell line; *p < .04, two-tailed t test). (C) Subcellular fractionation, followed by Western blot analysis, demonstrated platelet-dependent enrichment of VISTA expression, predominantly in the membrane compartment.

Our previous study demonstrated that platelets induce the immune checkpoint protein PD-L1 in ovarian cancer cells in vitro and in vivo.^7^ We investigated whether platelets affect VISTA expression in ovarian cancer cells. Co-incubation with washed platelets increased VISTA expression in several ovarian cancer cell lines (Figure 1A,B). We examined the increase in VISTA expression in the cytosolic, membrane, and nuclear compartments of ovarian cancer cells after exposure to platelets, using fractionation. Western blotting of various cellular fractions demonstrated that platelets predominantly increased VISTA expression in the cytosolic fraction, with minimal changes detected in the membrane fraction, and no detectable VISTA expression in the nuclear fraction (Figure 1C). These findings indicate that platelet-induced upregulation of VISTA primarily affects the cytosolic compartment. The original Western blots are shown in the Supplementary Figure 3.

### Platelets increased VISTA expression on monocytes and CD4+ T cells in the peripheral blood in mice

**Figure 2.**
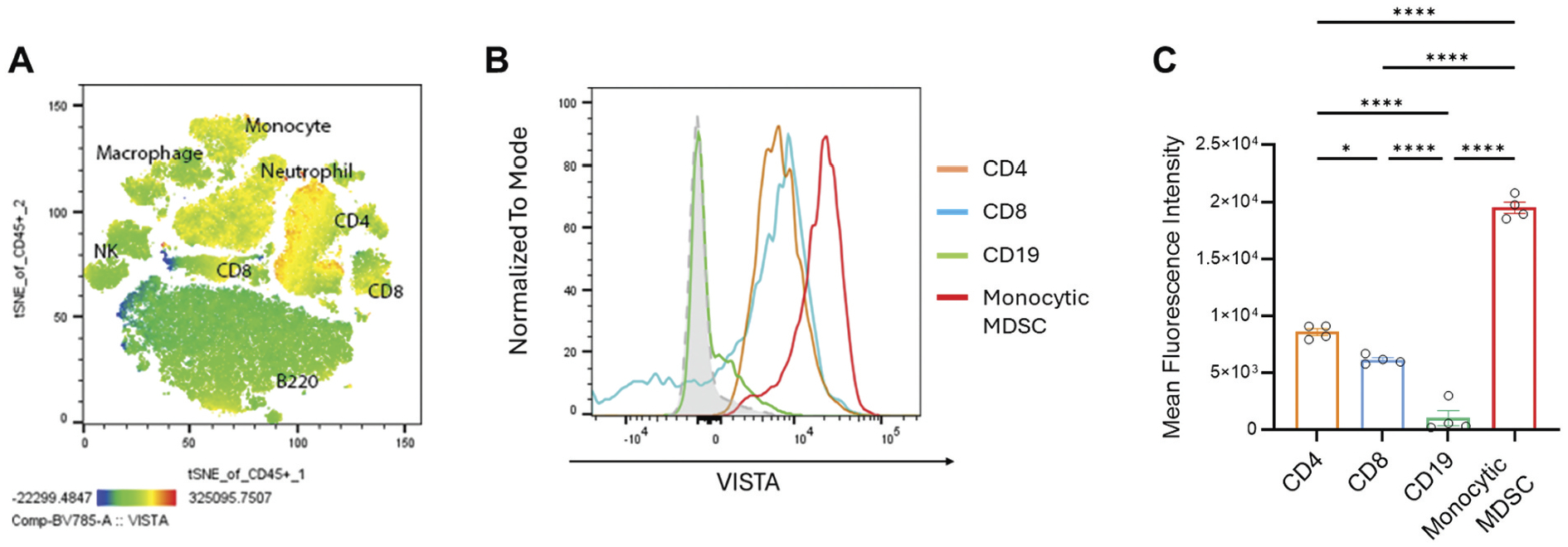
Expression of VISTA in mouse peripheral blood mononuclear cells (PBMCs). (A) t-distributed stochastic neighbor embedding (tSNE) visualization of CD45+ immune cells, highlighting distinct populations of myeloid cells and CD4+ T cells based on VISTA expression intensity. Color gradient indicates VISTA expression levels, ranging from low (blue) to high (red). (B) Histogram analysis of VISTA expression levels in specific PBMC subsets identified by lineage markers, B cells (CD19, green), CD4+ T cells (orange), CD8+ T cells (blue), and monocytes (CD14, red), and isotype control IgG (dashed gray). Data are representative of four independent experiments. (C) The average VISTA expression on immune cells from human peripheral blood was measured by flow cytometry and is presented as a bar graph of mean fluorescence intensity (*n* = 4 independent experiments; *p = .01, ***p < .0001, t-test, two-tailed).

To assess basal VISTA expression in immune cells, we performed flow cytometry and t-SNE analyses on peripheral blood mononuclear cells (PBMCs) from mice (Figure 2A). In the peripheral blood, CD14+ monocytes exhibited the highest levels of VISTA expression. In contrast, CD19+ B cells displayed little to no basal VISTA expression, and CD4+ and CD8+ T cells expressed a moderate amount of VISTA (Figure 2B,C).

**Figure 3.**
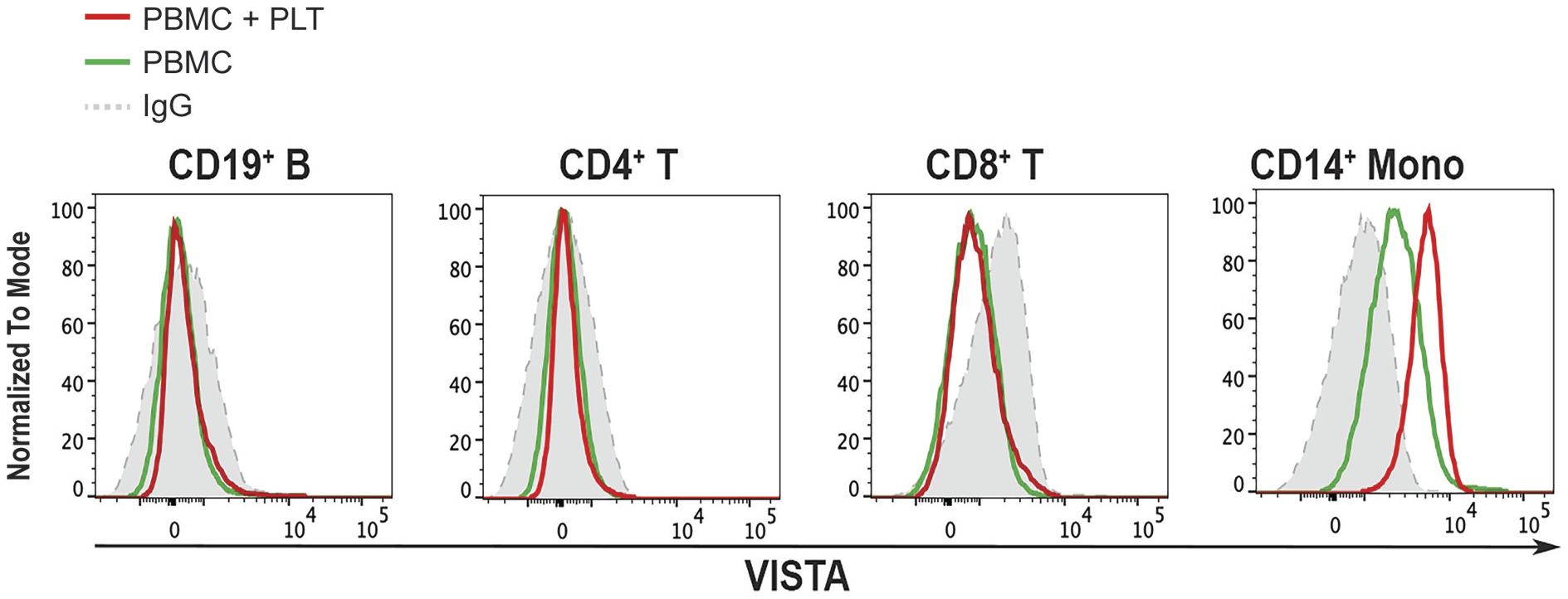
The effect of platelets on the expression of VISTA in the human peripheral blood mononuclear cells. representative histograms show staining intensity in CD19+ B cells, CD4+ T cells, CD8+ T cells, and CD14+ monocytes. Gray-filled histograms indicate unstained controls, green lines represent cells cultured without platelet co-incubation, and red lines represent cells cultured with platelet co-incubation. The data shown are normalized to the mode.

Upon co-incubation with platelets, CD14+ monocytes showed a marked upregulation of VISTA (Figure 3). This platelet-induced enhancement mirrored the basal distribution pattern, reinforcing that monocytes are the primary VISTA-expressing subset in the peripheral blood under both resting and platelet-stimulated conditions.

### High platelet counts increased VISTA expression in the tumor microenvironment

**Figure 4.**
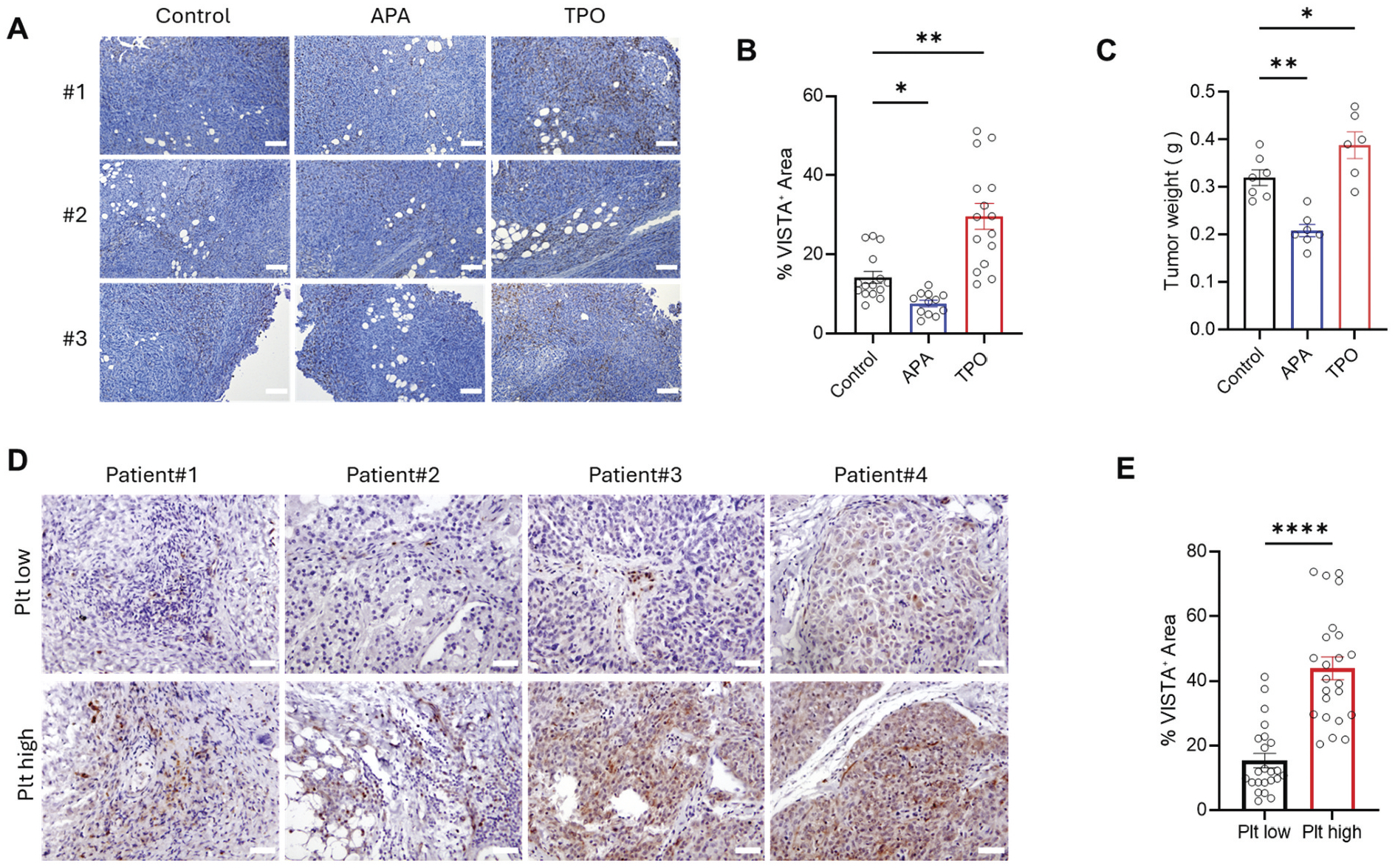
Immunohistochemical analysis illustrating platelet-dependent regulation of VISTA expression within tumor tissues (mouse and human). Representative images show anti-VISTA staining (brown) in tumor samples. (A) Tumors from control, antiplatelet agent (APA)-treated, and thrombopoietin (TPO)-treated mice. (B) VISTA expression in murine tumor specimens was quantified as the percentage of tissue surface area stained with anti-VISTA antibody on immunohistochemistry (IHC) slides (*n* = 12–30 mice/group, **p = .017, ***p < .0001. (C) Final tumor weight induced by ID8 murine ovarian cancer cells in control mice (0.32 g ± 0.04 g) and mice receiving antiplatelet antibodies (APA, 0.21 ± 0.03 g) or thrombopoietin (TPO, 0.39 ± 0.06 g). *n* = 6 mice per group. *p = .001, *p < .05. Scale bar, 100 μm. (D) Tumors from human ovarian cancer patients were categorized based on platelet counts, with tumors having low (<250,000/μL) and high (>450,000/μL) platelet counts. Increased VISTA expression correlates with elevated platelet counts. Scale bar: 100 μm. (E) VISTA expression in human tumor specimens was quantified as the percentage of tissue surface area stained with anti-VISTA antibody on immunohistochemistry (IHC) slides. Tumors from patients with high platelet counts expressed more VISTA as compared to patients with lower platelet counts (*n* = 23, ****p < .0001). All statistical analysis was performed with a two-tailed Student’s t-test. Scale bar, 100 μm.

We investigated how platelet levels influence VISTA expression in the tumor microenvironment (TME) by experimentally altering platelet counts in tumor-bearing mice. Immunohistochemical (IHC) analysis of tumors from untreated controls and mice treated with an antiplatelet antibody (APA) revealed that thrombocytopenia markedly reduced VISTA expression and was accompanied by diminished tumor growth.^7,26^ In contrast, administration of recombinant thrombopoietin (TPO), which elevated platelet counts, significantly increased VISTA expression relative to controls, correlating with enhanced tumor growth (Figure 4A–C).

To assess whether this relationship extends to human diseases, we analyzed ovarian cancer specimens stratified by patient platelet counts. Tumors from patients with low platelet counts (<250,000 platelets/μL) displayed relatively low VISTA expression, whereas tumors from patients with elevated platelet counts (>450,000 platelets/μL) exhibited significantly higher VISTA expression. These results indicate that, consistent with our murine findings, elevated platelet counts in human ovarian tumors are associated with increased VISTA expression (Figure 4D–E).

### VISTA expression in immune and stromal compartments of human ovarian cancer

**Figure 5.**
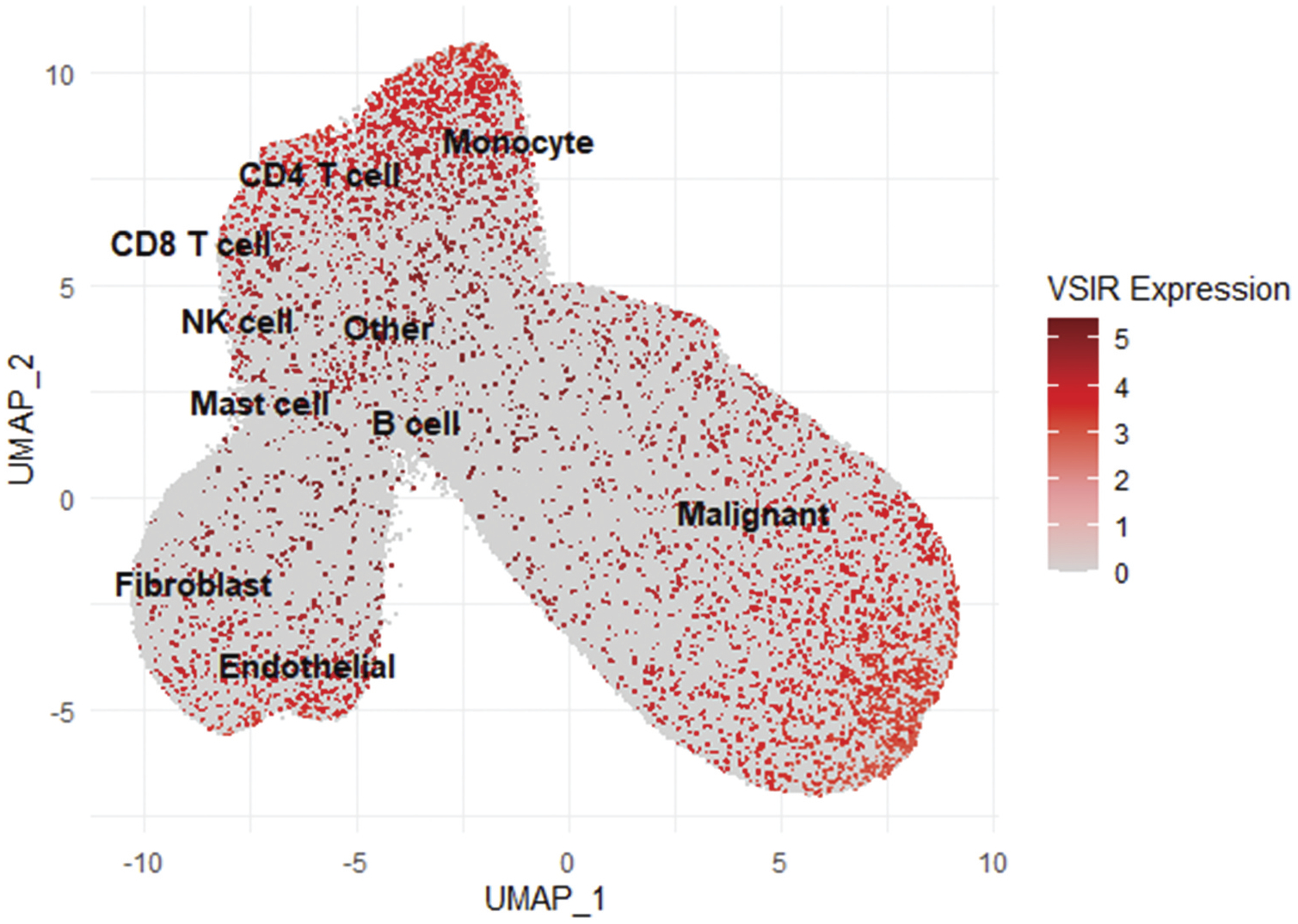
UMAP visualization of single-cell RNA sequencing from human ovarian cancer tissues demonstrating VISTA expression across immune and stromal subsets. Color intensity (red) indicates relative VISTA expression levels from low (gray) to high (dark red).

We characterized VISTA expression and spatial distribution within the tumor microenvironment (TME) of human serous ovarian carcinoma using single-cell RNA sequencing of tumor specimens. Uniform Manifold Approximation and Projection (UMAP) analysis demonstrated marked heterogeneity in VISTA expression among distinct cell populations. VISTA was most highly expressed in myeloid cells, with moderate expression in epithelial cancer cells and CD4+ lymphocytes, and low expression in endothelial cells and fibroblasts (Figure 5).

### VISTA expression in various tumors correlates with platelets

**Figure 6.**
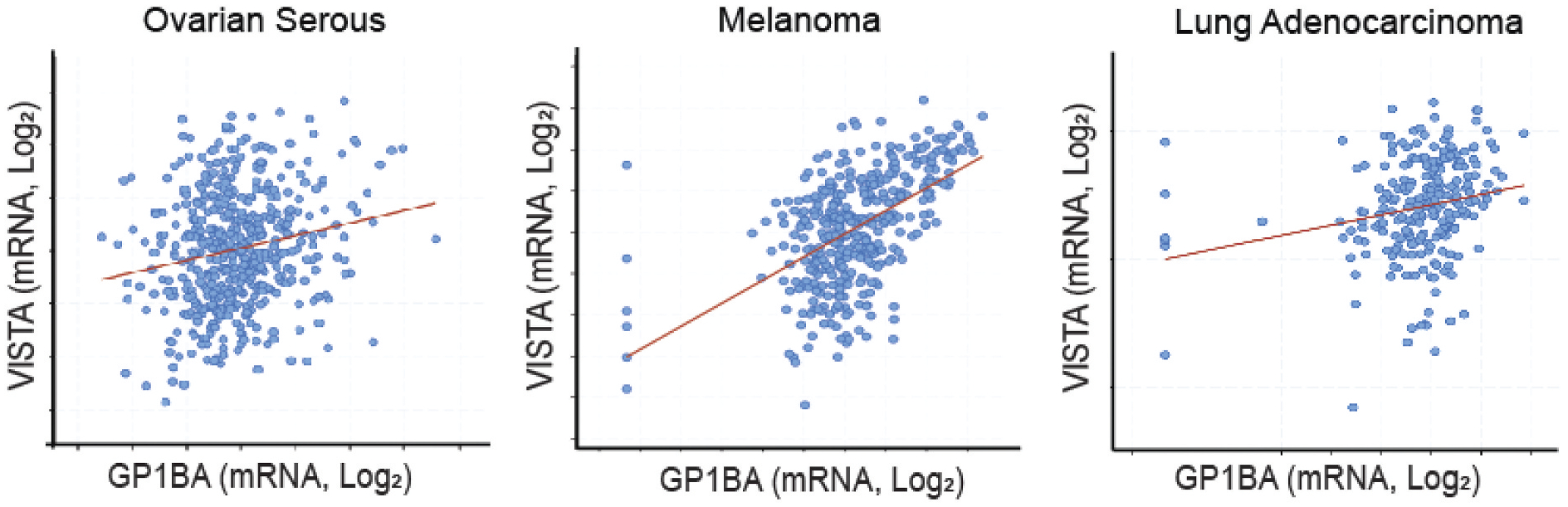
Correlation between *VSIR* (VISTA) and *GPIBA* (platelet glycoprotein Ibα) expression in human tumor samples (ovarian cancer, melanoma, and lung Adenocarcinoma).

We investigated the relationship between *GP1BA* (encoding platelet glycoprotein Ibα, predominantly expressed in megakaryocytes with remnant expression in platelets) and *VSIR* (encoding VISTA) expression in multiple human cancers, including ovarian cancer, melanoma, and lung adenocarcinoma. We performed correlation analyses between *GP1BA* and *VSIR* transcript levels using the Cancer Genome Atlas (TCGA) dataset. Scatter-plot analysis demonstrated a modest but consistent positive correlation (Pearson *r* ≈ 0.2; Log_2_-transformed values) across tumor types (Figure 6).

### Prognostic value of VSIR and GP1BA expression in ovarian cancer

**Figure 7.**
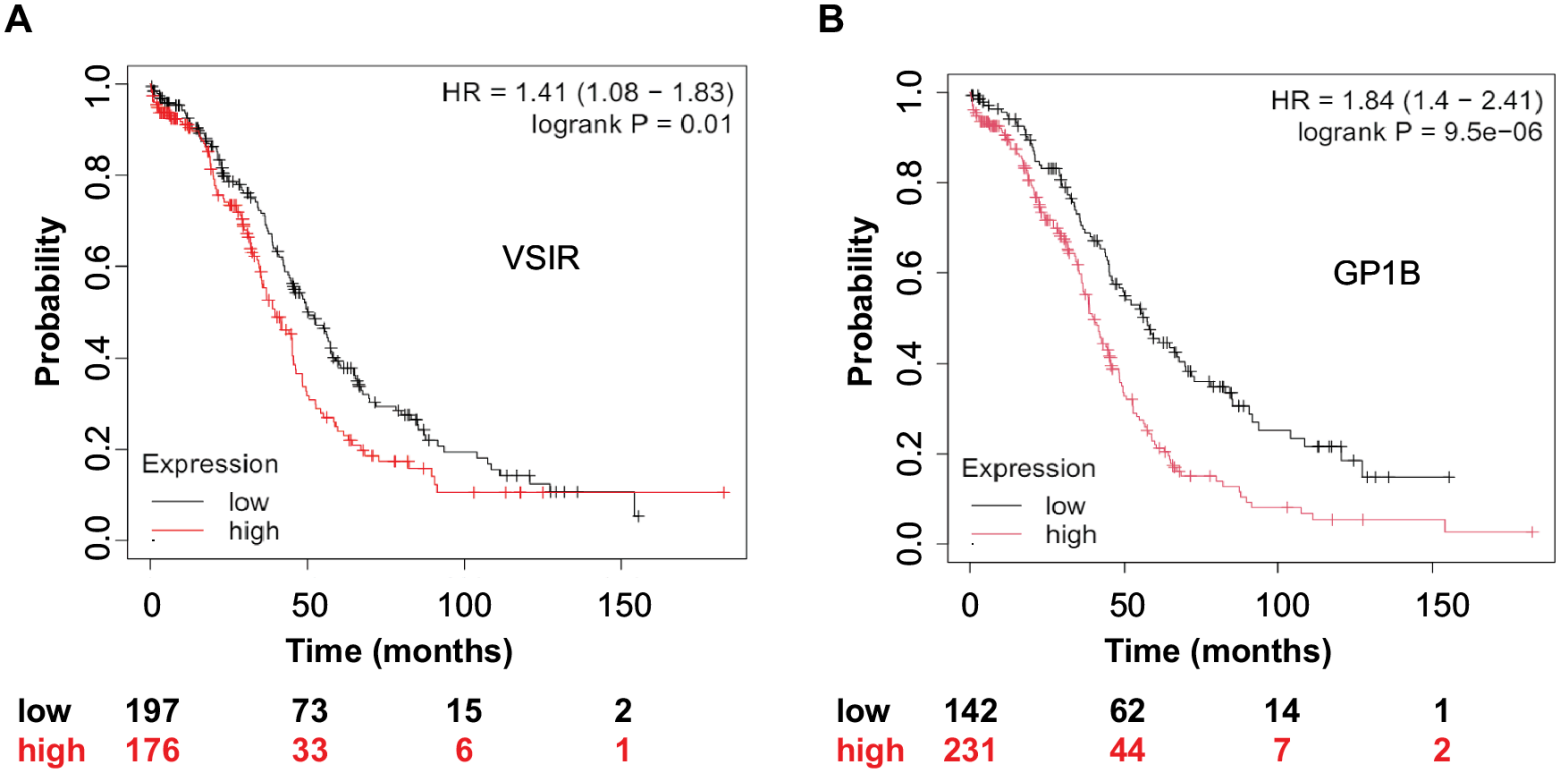
The expression of *VSIR* and *GPIBA* correlates with survival in ovarian cancer. The impact of VISTA expression and platelets on kaplan-meier survival curves in 374 patients with ovarian cancer.

We used publicly available datasets to identify and validate survival-associated genes in ovarian tumors.^30^ Kaplan–Meier analyses in a cohort of 374 patients with ovarian cancer revealed that both *VSIR* and *GP1BA* expression levels had prognostic significance (Figure 7). High *VSIR* expression was correlated with significantly reduced overall survival (hazard ratio [HR] = 1.41, 95% CI: 1.08–1.83; log-rank *p* = .01). Similarly, high *GP1BA* expression was associated with markedly worse prognosis (HR = 1.84; *p* < .0001).

### Impact of chemotherapy and anti-PD-L1 antibodies on VISTA expression in ovarian cancer

**Figure 8.**
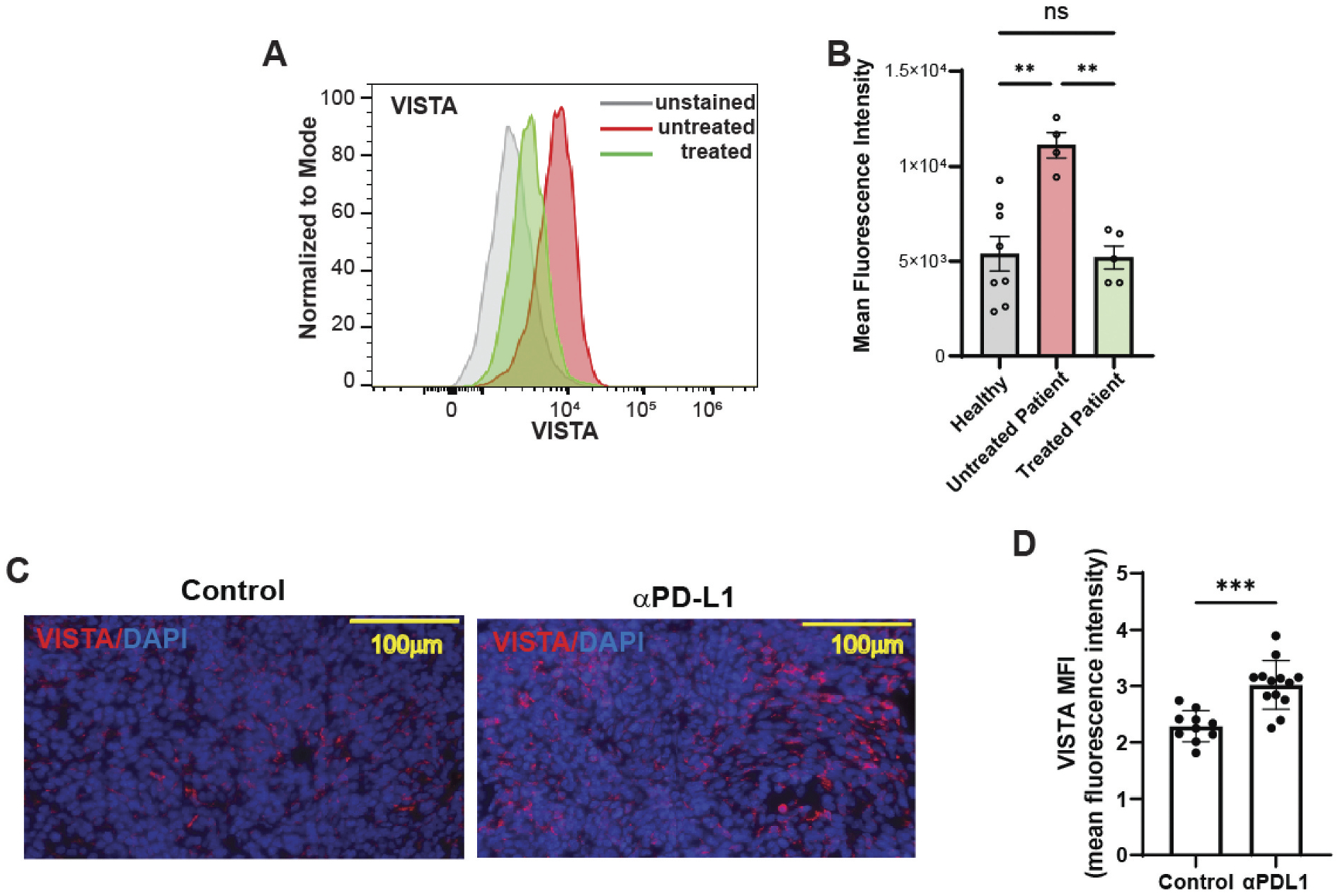
The impact of chemotherapy and anti-PD-L1 antibodies on VISTA expression. (A) The representative flow cytometry histogram of VISTA expression on CD14+ cells from the peripheral blood of five ovarian cancer patients before chemotherapy, four patients after chemotherapy, and eight control subjects. (B) Mean fluorescence intensity (MFI) quantification showing significant differences among conditions (one-way ANOVA followed by Turkey’s multiple comparison test, **p < .001). (C) Representative immunofluorescence images depicting VISTA expression (red) and nuclear staining (DAPI, blue) in tumor tissues from control or anti-PD-L1 antibody-treated (αPD-L1) ovarian cancer-bearing mice. Scale bar: 100 μm. (D) Qantification of mean fluorescence intensity (MFI) for VISTA expression from immunofluorescence images, demonstrating a significant increase in αPD-L1-treated samples compared to control. VISTA expression in ovarian cancer tumor specimens between control mice (*n* = 10) and mice treated with anti-PD-L1 antibodies (*n* = 13). Two-tailed Student’s t-test, **p = .01.

To evaluate the impact of chemotherapy on VISTA expression, we compared CD14^+^ monocytes isolated from the peripheral blood of five patients with ovarian cancer who had received neoadjuvant chemotherapy with those from four untreated patients. Chemotherapy was associated with a marked reduction in VISTA expression in the monocyte population (Figure 8A,B).

We next examined the effect of PD-L1 blockade on VISTA expression within the tumor microenvironment (TME) using murine ovarian tumor tissues treated with either an anti–PD-L1 antibody or an isotype control. Quantitative immunofluorescence analysis demonstrated a significant increase in VISTA mean fluorescence intensity (MFI) in anti–PD-L1–treated tumors compared to controls (*p = .01), indicating a potential compensatory upregulation of VISTA in response to PD-L1 inhibition (Figure 8C,D).

## Discussion

Traditionally recognized for their role in hemostasis, platelets have been increasingly implicated in cancer progression through their interactions with both tumor and immune cells. We and others have demonstrated that platelets promote murine ovarian tumor growth^23,25–27^ and modulate immune checkpoint pathways, including PD-L1, thereby influencing responses to anti–PD-L1 immunotherapy in both preclinical models and patients.^7^

In the present study, we identify a novel role for platelets in shaping the ovarian tumor microenvironment (TME) by enhancing immune suppression through upregulation of VISTA, an emerging cancer immunotherapy target known for its role in maintaining immune tolerance.^12^ In ovarian cancer, VISTA expression is more homogeneous and abundant than PD-L1.^14–17^ Notably, in our studies, anti–PD-L1 treatment in tumor-bearing mice increased VISTA expression, suggesting a compensatory mechanism that may facilitate immune escape following PD-L1 blockade.

Our findings demonstrated that platelets markedly increase VISTA expression in both tumor and immune cells, with the most pronounced effects observed in myeloid cells. In vitro co-culture experiments revealed that platelets induce robust VISTA upregulation in myeloid-derived suppressor cells (MDSCs) and CD4+ T cells, supporting a model in which platelets foster immune evasion by suppressing T-cell function while enhancing MDSC immunosuppressive activity. These observations are consistent with prior reports of platelet-mediated regulation of immune checkpoints^7^ and of immune evasion through shielding tumor cells from cytotoxic immune responses.^18–22^

A key insight from our study was the compensatory relationship between VISTA and PD-L1. This suggests that VISTA may sustain immune suppression even when PD-L1 expression is reduced, with important implications for immunotherapy resistance. As PD-L1 remains a central target of immune checkpoint inhibitors (ICIs), our data highlight the potential need for combinatorial blockade of both PD-L1 and VISTA to achieve optimal tumor control.

Clinically, high VISTA expression was associated with poor overall survival, underscoring its potential as a prognostic biomarker. However, patients with elevated VISTA levels showed an improved response to ICIs. This is particularly relevant in the context of thrombocytosis, a common feature in ovarian cancer, which may exacerbate VISTA-mediated immune suppression while also creating a therapeutic opportunity for immunotherapies.

The interplay between platelet-induced VISTA in immune cells and PD-L1 expression in tumor cells suggests that monotherapy against a single checkpoint may be insufficient to reverse immune suppression in the ovarian TME. Our results support the rationale for dual PD-L1/VISTA blockade, and further suggest that disrupting platelet–VISTA interactions via antiplatelet agents or direct VISTA inhibitors could be a promising adjunct to current immunotherapies. Future work should focus on elucidating the molecular pathways by which platelets regulate VISTA expression in monocytes and other immune cells and on testing combination regimens in preclinical and clinical settings, particularly in patients with high platelet counts.

Immune checkpoint expression in the ovarian tumor microenvironment is dynamically regulated by both tumor-intrinsic factors and extrinsic signals from stromal and immune cells, including platelets. In ovarian cancer, PD-L1 expression on tumor cells is upregulated by platelets through both contact-dependent NF-κB signaling and contact-independent TGF-βR1/Smad pathways, thereby enhancing immune evasion and blunting antitumor T-cell responses.^7^ Elevated platelet counts correlate with increased PD-L1 expression in both murine and human ovarian tumors, and preclinical models show that thrombocytosis can enhance the efficacy of anti–PD-L1 therapy. At the same time, antiplatelet agents may reduce their benefit.^7^ In this context, our finding that platelets also induce the immune checkpoint VISTA in ovarian cancer cells and myeloid cells adds another layer of immune modulation: platelets promote a broadly immunosuppressive TME by coordinately upregulating multiple inhibitory checkpoints. The compensatory upregulation of VISTA following PD-L1 blockade further suggests that tumors may exploit alternative checkpoints to maintain immune suppression, highlighting the potential need for dual targeting of PD-L1 and VISTA, particularly in patients with high platelet counts who exhibit heightened checkpoint expression.

Although our data establish that platelets are potent inducers of VISTA expression in myeloid and tumor cells, the molecular mechanisms underlying this effect remain to be fully defined. Platelets contain a diverse repertoire of immunoregulatory mediators, including TGF-β, CD40L, PF4, RANTES, and other cytokines and growth factors, that can modulate transcriptional programs in monocytes and tumor cells through NF-κB, Smad, and STAT-dependent signaling pathways. It is plausible that one or more of these soluble factors contribute to VISTA upregulation, either directly by activating transcription of the VSIR gene or indirectly by driving myeloid cell differentiation toward a more suppressive phenotype. In addition, platelets engage immune and tumor cells via multiple surface receptors, including P-selectin, integrins, and CLEC-2, suggesting that contact-dependent signaling also contributes to VISTA induction. Dissecting the relative contribution of these pathways will require systematic evaluation of platelet releasates and receptorblocking approaches in future studies. An additional variable that may influence this process is platelet heterogeneity. Reticulated (immature) platelets are larger, more transcriptionally and metabolically active, and contain greater amounts of RNA and granule cargo than older circulating platelets. These young platelets exhibit enhanced reactivity and increased protein synthetic capacity, features that could amplify their ability to deliver immunomodulatory signals and drive checkpoint expression. Patients with cancer, particularly those with thrombocytosis, often exhibit an expanded reticulated platelet fraction, suggesting that the balance between immature and mature platelets may determine the intensity of VISTA induction in the tumor microenvironment. Whether reticulated platelets preferentially promote VISTA expression compared with older platelets is an important unanswered question.

In conclusion, our study revealed a previously unrecognized role for platelets in promoting an immunosuppressive TME via VISTA upregulation and in modulating PD-L1–VISTA compensatory dynamics. Given the prevalence of thrombocytosis in ovarian cancer and its impact on immune checkpoint regulation, integrating platelet-targeted strategies with immune checkpoint blockades represents a compelling avenue for overcoming therapeutic resistance and improving patient outcomes.

## Supplementary Material

Supp 1

Supplemental data for this article can be accessed online at https://doi.org/10.1080/09537104.2026.2644366

## Data Availability

Research Data supporting this publication are available from the corresponding authors upon request.

## PLAIN LANGUAGE SUMMARY

### What is the context?

Ovarian cancer is a leading cause of death from gynecologic cancers. The tumor microenvironment (TME) in ovarian cancer is strongly immunosuppressive, limiting the success of current immunotherapies. Platelets, known for their role in blood clotting, are increasingly recognized as contributors to cancer growth and immune evasion. They can interact with tumor and immune cells, releasing molecules that suppress immune responses. VISTA, a newer immune checkpoint molecule, is widely expressed in ovarian cancer and may act as a key regulator of tumor-induced immune suppression.

### What is new?

This study reveals that platelets increase the expression of VISTA in both ovarian cancer cells and immune cells, such as monocytes. In mouse models, high platelet counts led to increased VISTA expression and faster tumor growth, while platelet depletion reduced both. Human ovarian cancer samples from patients with elevated platelet counts also showed higher VISTA levels. Blocking PD-L1, another immune checkpoint, led to increased VISTA expression, suggesting that tumors may use VISTA to bypass PD-L1–directed therapies.

### What is the impact?

These findings identify a new mechanism by which platelets promote immune suppression in ovarian cancer. VISTA may help tumors evade immune attack, particularly in patients with high platelet counts. Targeting both platelet activity and VISTA could enhance the effectiveness of immunotherapies and improve outcomes for patients with ovarian cancer.
