# Supplementary material for: Platelets induce VISTA expression and modulate the ovarian tumor microenvironment
Source: Platelets. Author manuscript; Available in PMC 2026 Jun 3. (PMC13231984; doi:10.1080/09537104.2026.2644366)

**Supplementary Table 1**

| **In vivo Experiment** |  |  |  |  |
| --- | --- | --- | --- | --- |
| **Reagent** | **Vendor** | **catalog No.** | **RRID** |  |
| C57/B6 | The Jaxson Laboratory | 664 | IMSR_JAX:000664 |  |
| Mouse TPO recombinant protein | PeProTech | 315-14-50UG |  |  |
| Anti-platelet antibody | Emfret | R300 | AB_2721041 |  |
| Anti-PD-L1 | Bio X Cell | BE0101 | AB_10949073 |  |
| IgG2b isotype control | Bio X Cell | BE0090 | AB_1107780 |  |
|  |  |  |  |  |
| **Antibodies** |  |  |  |  |
| **Reagent** | **Vendor** | **Catalog** | **RRID** | **For use** |
| anti-hu FITC-CD3 | Biolegend | 317306 | AB_571907 | Flow Cytometry |
| anti-hu PerCP-CD45 | Biolegend | 368506 | AB_2566358 | Flow Cytometry |
| anti-hu Alexafluor405-VISTA | R&D systems | FAB71267V | AB_3652260 | Flow Cytometry |
| anti-hu PE-Cy7-CD11b | Biolegend | 393103 | AB_2734450 | Flow Cytometry |
| anti-hu APC-CD19 | Biolegend | 302212 | AB_314242 | Flow Cytometry |
| anti-hu APC-Cy7 CD16 | Biolegend | 302018 | AB_314218 | Flow Cytometry |
| anti-hu PE-CD14 | Biolegend | 301806 | AB_314188 | Flow Cytometry |
| anti-hu PE-Dazzle594-PD-L1 | Biolegend | 124324 | AB_2565639 | Flow Cytometry |
| anti-hu PerCP-Cy5.5-CD41a | Biolegend | 303720 | AB_2561731 | Flow Cytometry |
| Ghost DyeUV450 | Tonbo | 13-0868-T500 |  | Flow Cytometry |
| anti-mouse PE/CF594-CD45 | Biolegend | 103149 | AB_2564590 | Flow Cytometry |
| anti-mouse APC/Cy7-CD3 | Biolegend | 100222 | AB_2242784 | Flow Cytometry |
| anti-mouse BV421-CD4 | Biolegend | 100563 | AB_2563052 | Flow Cytometry |
| anti-mouse PE/Cy7-CD8 | BD Biosciences | 552877 | AB_394506 | Flow Cytometry |
| anti-mouse BUV737-B220 | BD Biosciences | 612839 | AB_2870161 | Flow Cytometry |
| anti-mouse FITC- Ly-6C | Biolegend | 128005 | AB_1186134 | Flow Cytometry |
| anti-mouse BUV395-Ly-6G | BD Biosciences | 563978 | AB_2716852 | Flow Cytometry |
| anti-mouse PE/Cy5-F4/80 | Biolegend | 123112 | AB_893482 | Flow Cytometry |
| anti-mouse BV605-CD11b | Biolegend | 101257 | AB_2565431 | Flow Cytometry |
| anti-mouse APC-CD11c | Biolegend | 117310 | AB_313779 | Flow Cytometry |
| anti-mouse PE-NK1.1 | Biolegend | 108707 | AB_313394 | Flow Cytometry |
| anti-mouse BV786-VISTA | BD Biosciences | 742725 | AB_2741001 | Flow Cytometry |
| PE-labeled Rat Anti-Mouse Integrin αIIbβ3 (JON/A) | emfret | M032-2 | AB_2833084 | Flow Cytometry |
| VISTA (D5L5T) | Cell signaling | 54979 | AB_2799474 | Western blot/IF/IHC |
| Lamin A/C | Cell signaling | 4777 | AB_10545756 | Western blot |
| Na/K-ATPase | Santa Cruz biotechnology | sc-21712 | AB_626713 | Western blot |
| GAPDH | Santa Cruz biotechnology | sc-47724 | AB_627678 | Western blot |
|  |  |  |  |  |
| **Reagent** |  |  |  |  |
| **Reagent** | **Vendor** | **Catalog** | **RRID** |  |
| Hank's balanced salt solution (HBSS) | GenDEPOT | CA047 |  |  |
| Acid Citrate Dextrose (ACD) | Sigma-Aldrich | C3821 |  |  |
| RPMI-1640 | GenDEPOT | CM058 |  |  |
| DMEM | GenDEPOT | CM002 |  |  |
| Fetal Bovine Serum | GenDEPOT | F0901 |  |  |
| Penicillin-Streptomycin | GenDEPOT | CA005 |  |  |
| Dulbecco's Phosphate-Buffered Saline (DPBS) | GenDEPOT | CA008 |  |  |
| Ficoll Paque plus | Cytiva | 17144002 |  |  |
| Acridine Orange/Propidium Iodide stain | Logos biosystems | F23001 |  |  |
| 16% paraformaldehyde | Electron Microscopy Seicence | 15710 |  |  |
| VECTASTAIN® ABC-HRP Kit, Peroxidase | Vector Laboratories | PK-4001 | AB_2336819 |  |
| DAB Substrate Kit, Peroxidase (HRP) | Vector Laboratories | SK-4110 | AB_2336820 |  |
| RIPA | Milipore Sigma | R0278 |  |  |
| Protease inhibitor | Roche | 11836170001 | AB_2915941 |  |
| BCA protein assay | Thermo Scientific | 23227 |  |  |
| Blocking solution | Bio-Rad | 1706404 |  |  |
| Anti-rabbit IgG, HRP | Cell signaling | 7074 | AB_2099233 |  |
| anti-Goat IgG, HRP | Invitrogen | 31402 | AB_228356 |  |
| Anti-mouse IgG, HRP | Cell signaling | 7076 | AB_330924 |  |
| Donkey serum | Sigma-Aldrich | D9663 | AB_2337258 |  |
|  |  |  |  |  |
| **Software** |  |  |  |  |
| **Reagent** | **Vendor** |  | **RRID** |  |
| Single cell portal | https://singlecell.broadinstitute.org/single_cell | | SCR_014816 |  |
| FlowJo v10.10 | Tree Star |  | SCR_008520 |  |
| GraphPad Prism v10.6 | GraphPad Software Inc. |  | SCR_002798 |  |
| Fiji | National Institutes of Health |  | SCR_002285 |  |
| R v4.5.0 | R Project |  | SCR_001905 |  |
| Seurat | Seurat |  | SCR_016341 |  |
| ggplot2 | CRAN |  | SCR_014601 |  |
| Cytek Aurora Spectral Analyzer | Cytek Biosciences |  | SCR_019826 |  |
|  |  |  |  |  |

**Supplementary Figure1**

**
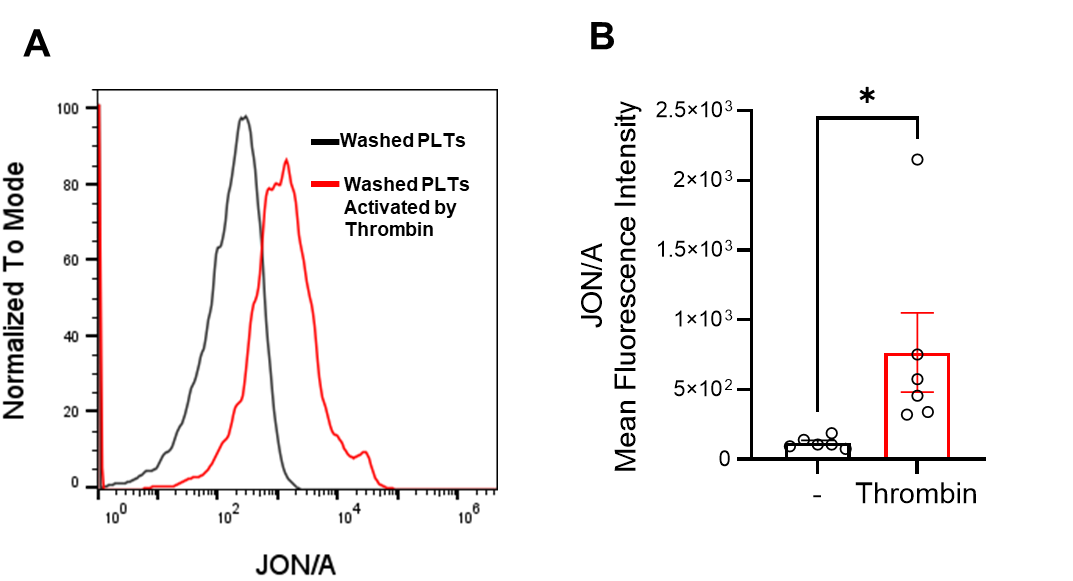
**

**Supplementary Figure1: Binding of the Jon/A antibody (recognizing active conformation of GPIIb-IIIa) on the surface of washed platelets before and after exposure to thrombin.** Washed murine platelets were incubated with anti-mouse JON/A-PE, and then thrombin (0.1U/ml) was added to initiate activation. Samples were incubated for 20 minutes at room temperature. The reaction was stopped by fixation with 1% paraformaldehyde. Data were acquired using Cytek Aurora spectral cytometer. **(A)** Histograms of a single flow cytometry experiment. **(B)** The average of six independent flow cytometry experiments is shown in the graphs (* p<0.05, two-tailed Student's t-test).

**Supplementary Figure 2**

1. **Gate strategy for Figure 2A**

**
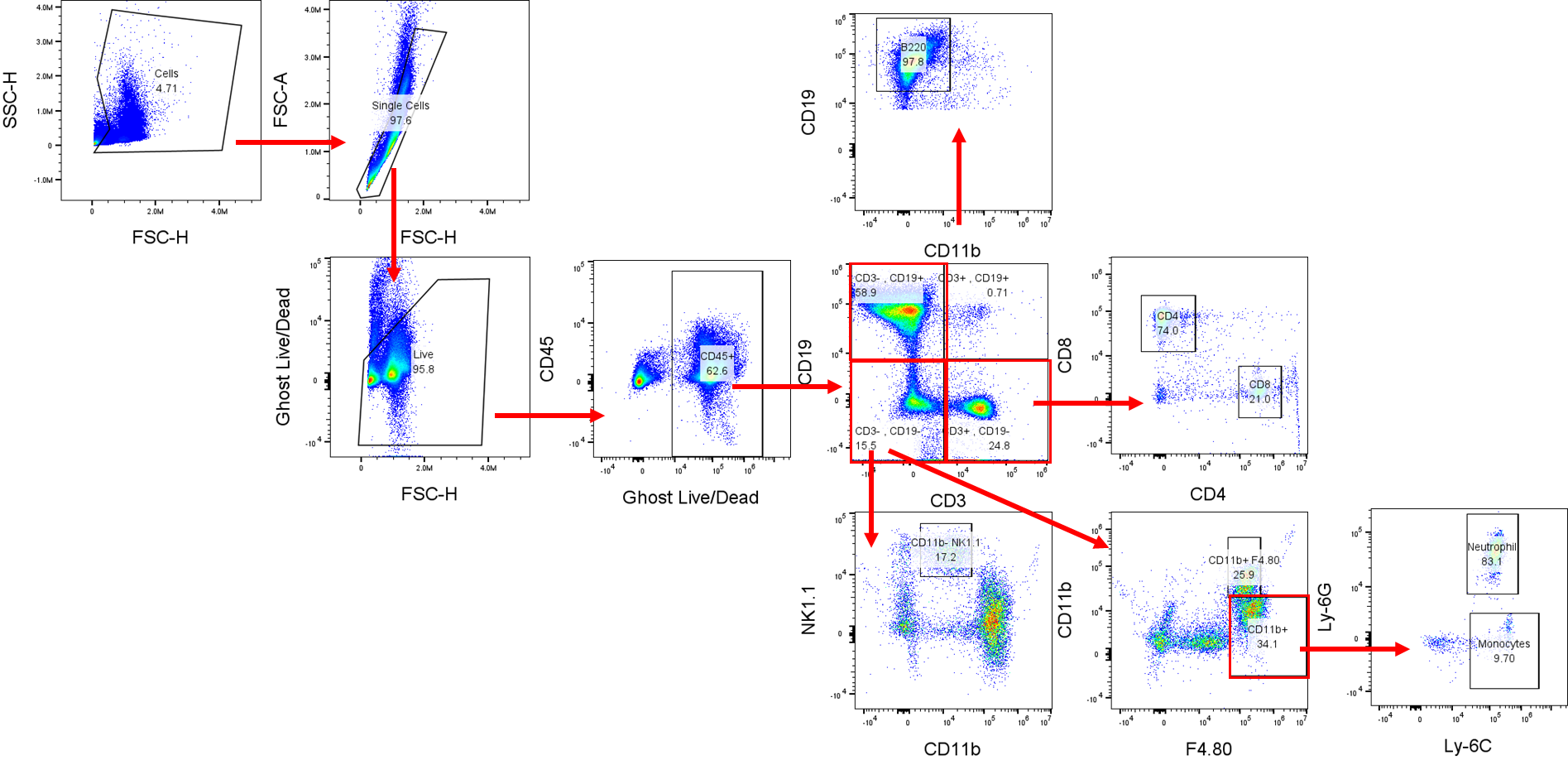
**

1. **Gate strategy for Figure 2B-C**

**
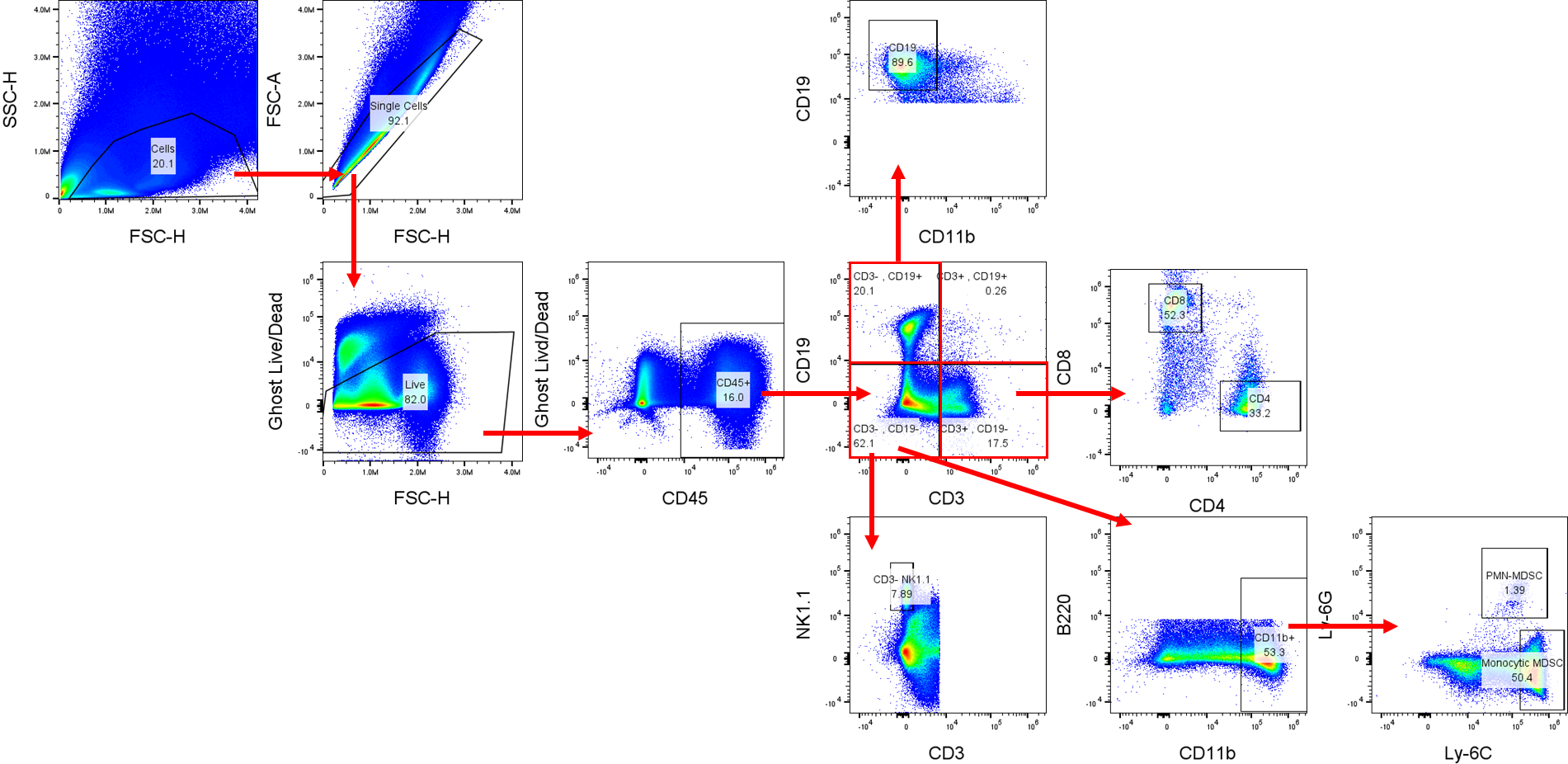
**

1. **Gate strategy for Figure 3**

**
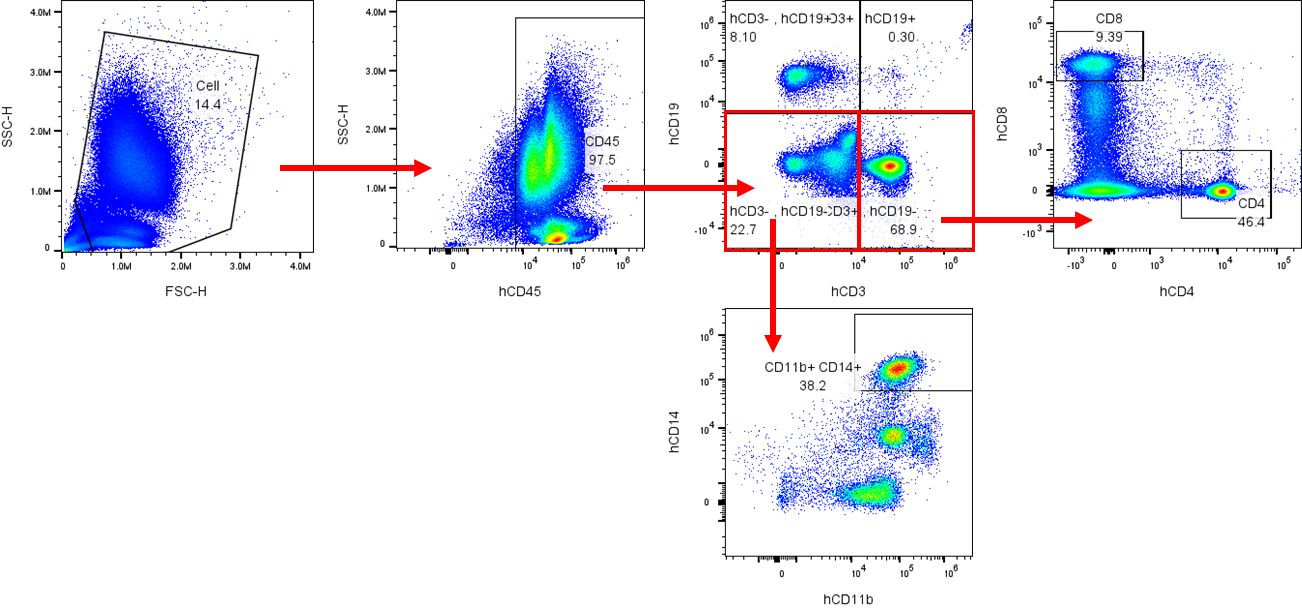
**

**Supplementary Figure3**
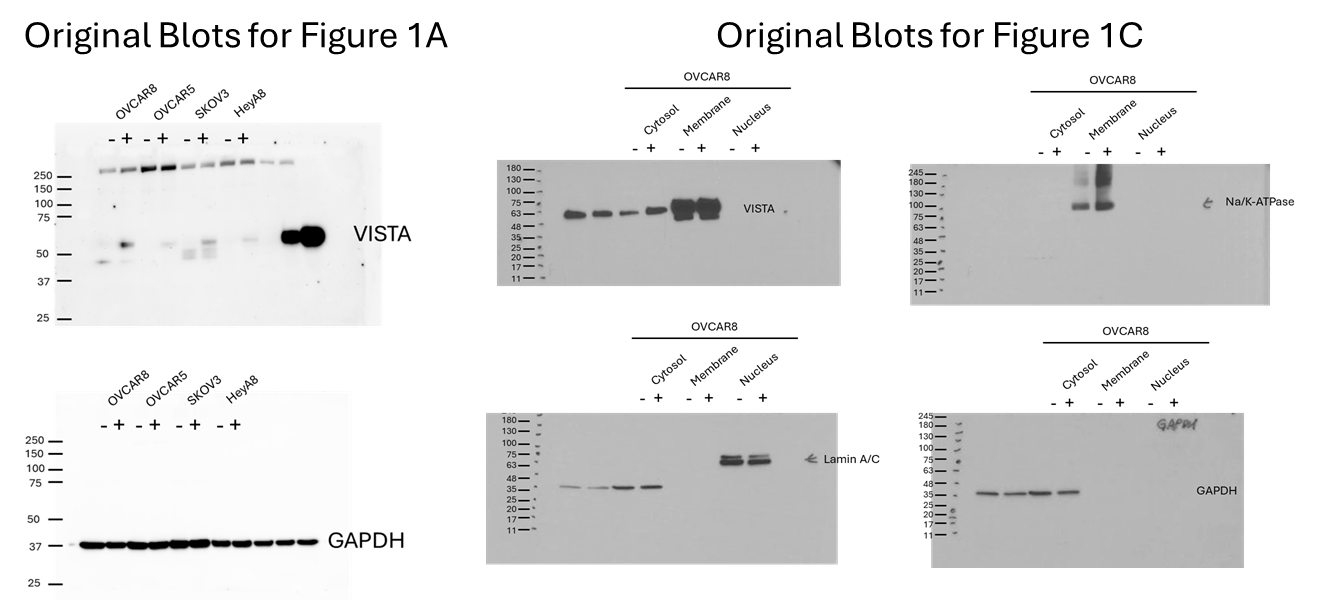

Supplement: Supp 1 [file NIHMS2161669-supplement-Supp_1.docx]
